# Root Coverage for Single Deep Gingival Recessions: Outcomes Based on a Decision-Making Algorithm

**DOI:** 10.1155/2019/1830765

**Published:** 2019-01-22

**Authors:** João B. César Neto, Marília C. Cavalcanti, Ricardo T. Sekiguchi, Claudio M. Pannuti, Giuseppe A. Romito, Dimitris N. Tatakis

**Affiliations:** ^1^Division of Periodontology, College of Dentistry, University of São Paulo, São Paulo, Brazil; ^2^Division of Periodontology, College of Dentistry, The Ohio State University, Columbus, OH, USA

## Abstract

**Aim:**

The aim of this study is to report root coverage outcomes in single deep gingival recessions (GR) following a proposed decision-making algorithm.

**Materials and Methods:**

A retrospective, practice-based study included single deep (≥5 mm) Miller Class II and III defects. The step-by-step decision-making algorithm led to a choice among three different flap designs (coronally advanced flap (CAF), double papilla envelope flap (DPE) or modified lateral sliding flap (LSF)) used with a connective tissue graft. Recession depth (RD) at 6 months follow-up and the corresponding root coverage (RC) were the primary outcomes assessed.

**Results:**

Sixteen GR defects were included, with baseline RD of 6.7 ± 1.8 mm. Six months postoperatively, RD was significantly reduced to 1.2 ± 0.8 mm (*p* < 0.05). Mean RC was 81.7 ± 13.0%, without significant differences between Miller Class II (87.1 ± 9.2%; *n*=9) and Class III (74.6 ± 14.5%; *n*=7) GRs (*p*=0.07). Postoperatively, keratinized tissue width increase was greater for LSF (3.5 ± 1.1) and DPE (4.2 ± 1.4 mm) than for CAF (1.9 ± 0.9 mm).

**Conclusions:**

Following the proposed decision-making algorithm, root coverage outcomes for GR defects ≥5 mm were comparable to outcomes reported for shallow defects. Prospective clinical trials are needed to validate the proposed approach and techniques.

**Practical Implications:**

The proposed algorithm allows the clinician to select the appropriate surgical technique for treatment of single deep gingival recessions with good predictability.

## 1. Introduction

Gingival recession (GR) is a common periodontal condition that can negatively impact esthetics, plaque control, and hypersensitivity [1]. Furthermore, the exposed root surfaces are susceptible to root caries and noncarious cervical lesion development [2, 3]. In patients with good oral hygiene, longitudinal evidence indicates that untreated GR defects tend to increase in depth over time [4].

The outcomes of available GR treatment modalities have been analyzed through several systematic reviews, which conclude that use of subepithelial connective tissue graft (CTG) provides the best results for predictable and long-lasting root coverage (RC) [5–7]. However, GR treatment outcomes may be modulated by defect characteristics, such as defect dimensions (depth, width), site (maxilla, mandible), defect number (single, multiple), soft tissue anatomy (keratinized tissue quality/quantity; papilla height/width; frenum/muscle pull; vestibular depth), and tooth position [7–10]. Despite the extensive literature on GR treatment, adequate evidence is lacking on outcomes at specific sites other than maxillary canines and premolars or on the effect of site characteristics, such as root prominence and vestibular depth [9].

Most of the existing literature has explored treatment of 2–4 mm deep GRs, providing limited evidence on deeper defects. Evidence indicates that deeper GR defects represent more of an aesthetic concern for patients and is one of the reasons for which they seek treatment [11]. The definition of a “deep” GR defect varies widely in the literature: authors have used subjective assessment [12], depth ≥3 mm [13–15], >3 mm [16], ≥4 mm [10, 17–19], ≥5 mm [20], or >5 mm [21, 22]. Despite the lack of consensus on the definition of a “deep” GR defect, evidence indicates that increasing GR depth negatively affects RC outcomes [7]. To overcome the challenges of treating deep GR defects, diverse CTG-based surgical approaches have been proposed; these combine CTG with envelope flap or lateral sliding flap (LSF) or coronally advanced flap (CAF) [21]. Close attention to confounding anatomical factors, such as increased defect width and shallow residual vestibular depth, is needed when dealing with deep GR defects [21]. Therefore, the decision-making process for treatment of deep GR defects is complex, requiring careful consideration of additional anatomical parameters.

The aim of this retrospective, practice-based case series study is to evaluate the outcomes of treating deep (≥5 mm) GR defects when following a step-by-step clinical decision-making algorithm which incorporates assessment of GR dimension, residual vestibular depth, and potential compromising factors.

## 2. Materials and Methods

### 2.1. Patient and Site Selection

The records of adult and systemically healthy patients who were referred to a private periodontal practice (Sorocaba-SP, Brazil) for GR evaluation and treatment were reviewed. Patients treated between October 2009 and May 2016 for a single deep (≥5 mm in depth) and Miller Class II or III [23] defect were included. All patients were given detailed information on surgical procedures, materials, medications, anticipated outcomes, potential complications, side effects, and alternative treatment options; all provided informed consent prior to surgery. Eligibility criteria were adult (≥18 years old), nonsmoker, nonsignificant and noncontributory medical history, no systemic medications, periodontally healthy, single deep GR defect treated, at least 6-months of postoperative follow-up, and documentation of clinical parameters reported below. The study protocol was approved by the Ethics Review Board of College of Dentistry (USP) (ERB approval n. 1.981.731).

### 2.2. Preoperative Patient and Site Management

Patients received oral hygiene instructions, prophylaxis, or scaling and root planing, as needed, prior to surgery. Surgical procedures were not scheduled until patients achieved satisfactory oral hygiene levels (plaque score <20%, plaque-free GR site and adjacent teeth, and negative for bleeding on probing (BOP)).

### 2.3. Clinical Parameters

Clinical parameters were recorded immediately preoperatively (baseline) and six months postoperatively (Tables 1 and 2). Evaluated parameters were plaque score; BOP; recession depth (RD; cementoenamel junction (CEJ) to gingival margin (GM)); recession width (RW) at the CEJ level; GR classification [23]; probing depth (PD); clinical attachment level (CAL); and keratinized tissue width (KTW; GM to mucogingival junction (MGJ)). Plaque and BOP were recorded at 6 sites per tooth on all teeth present. The remaining parameters were measured on the midbuccal aspect of the study tooth and recorded to the nearest 0.5 mm. Parameters were measured using a periodontal probe (UNC-15 probe, Hu-Friedy, Chicago, IL, USA) by the same operator (JBCN) who performed the procedures.

Root coverage (RC, in percentage) was calculated by the following formula:

(1) $$\begin{array}{c} \left(\frac{\text{baseline RD}-6\text{-months RD}}{\text{baseline RD}}\right)\times 100. \end{array}$$

### 2.4. Decision Tree

**Figure 1:**
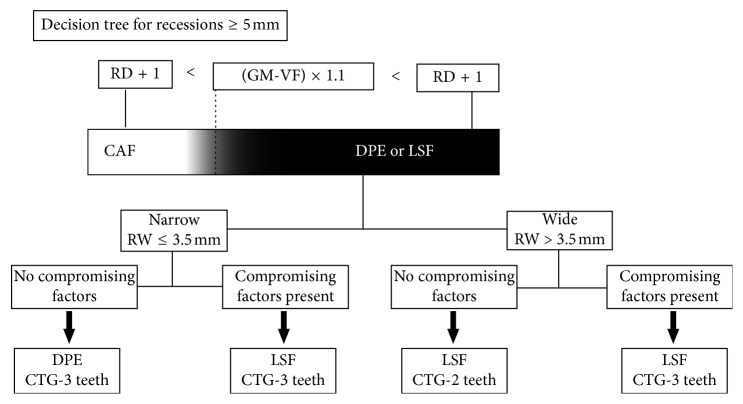
Graphic illustration of the decision-making process. When the GM-VF distance is clearly greater than RD, CAF + CTG should be adopted. In borderline cases (gray color), CAF + CTG was adopted. When CAF is rejected, narrow recessions should be treated with DPE + CTG and wide recessions should be treated with LSF + CTG. The presence of compromising factors in narrow defects moves the decision towards LSF + CTG. In addition, 3-teeth length is recommended for CTG in the presence of compromising factors (see text for extended explanation). RD = recession depth; GM = gingival margin; VF = vestibule fornix; CAF = coronally advanced flap; DPE = double papilla envelope; LSF = lateral sliding flap; CTG = connective tissue graft; RW = recession width.

**Figure 2:**
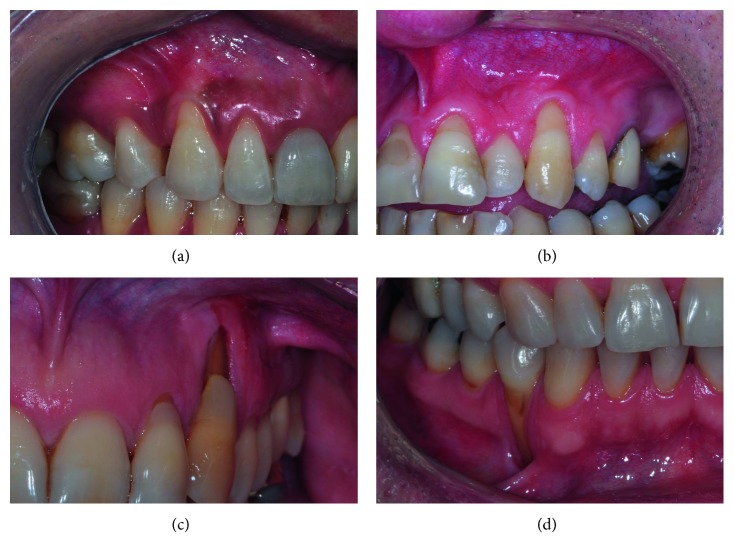
Clinical images illustrating the relationship between RD and GM-VF distance. From (a–d), it is possible to note that as RD increases, the GM-VF distance decreases. This affects the availability of tissue to displace coronally and the possible muscular tension on the flap. For abbreviations, see Figure 1 caption.

In the course of treating deep GR defects, the primary author (JBCN) has been following a defined step-by-step decision-making algorithm (Figure 1). The first step is to determine the anticipated GM displacement necessary to cover the defect in relation to the remaining vestibular depth. The decision tree considers that the necessary GM displacement should allow RD coverage and postoperative GM positioning 1 mm coronal to CEJ; therefore, the anticipated GM displacement (in mm) equals RD + 1. The relation between anticipated GM displacement and residual vestibular depth modulates flap design choice. This is because as RD increases, the distance between GM (RD site) and vestibular fornix (VF) depth (GM-VF distance) decreases, thus resulting in reduced tissue length available for coronal displacement (Figure 2). Hence, a formula was created to facilitate interpretation of the clinical parameters that guide the decision-making.

To measure the GM-VF distance, a lip retractor was used to gently retract the tissues to allow visualization of the VF. Meanwhile, the patient was asked to keep the mouth slightly open (about 10 mm), to avoid tension on the lower lip. Then, the periodontal probe, positioned along the long axis of the tooth and resting against the buccal surface of the tooth/alveolar process, was used to measure the distance from GM to VF. To determine when the probe tip touched the VF, the probe was viewed from an approximate 45° horizontal angle relative to the buccal surface of the tooth.

When RD + 1 < (GM-VF) × 1.1, a coronally advanced flap plus CTG (CAF + CTG) design was used (Figure 1). Otherwise, alternative designs were adopted. In borderline cases, where the flap design choice was deemed ambivalent, the CAF design was chosen. The use of the multiplication factor (×1.1) ensures that the remaining buccal tissue height (GM-VF) is at least 10% greater than the anticipated gingival margin displacement (RD + 1), a needed condition that became apparent from working on and analyzing cases of RD ≥ 5 mm.

Whenever CAF + CTG was rejected, the choice of flap design was either double papilla envelope flap (DPE + CTG) or lateral sliding flap (LSF + CTG) (Figure 1). The second step of the decision tree was to choose between DPE and LSF (Figure 1). The primary factor determining this decision was RW, classified as narrow (≤3.5 mm) or wide (>3.5 mm) [24, 25]. DPE was employed in narrow defects, and LSF was chosen for wide defects (Figure 1). This decision step was modified by additional factors, considered as potentially compromising the treatment outcome. Such factors included buccal tooth position, root prominence, proximity of vital structures (e.g., mental nerve), and deep bone dehiscence. The presence of compromising factors led to LSF use even in narrow GR defects (Figure 1).

The third step in the decision process was concerning CTG length (mesiodistal dimension), which varied by flap design and compromising factor presence (Figure 1). With CAF + CTG approach, CTG length equaled RW + 6 mm (3 mm on either side of the GR). With DPE + CTG approach, CTG length equaled RW plus width of two adjacent teeth. With LSF + CTG choice, CTG length varied depending on the absence/presence of compromising factors; in the absence of compromising factors, CTG length equaled RW plus width of one adjacent tooth (the tooth associated with the tunnel). When compromising factors were present, CTG length equaled RW plus width of two adjacent teeth. In all cases, CTG height (apicocoronal dimension) was as close to RD as possible; when donor site dimensions permitted, CTG height was RD + 2 mm.

### 2.5. Surgical Protocols

**Figure 3:**
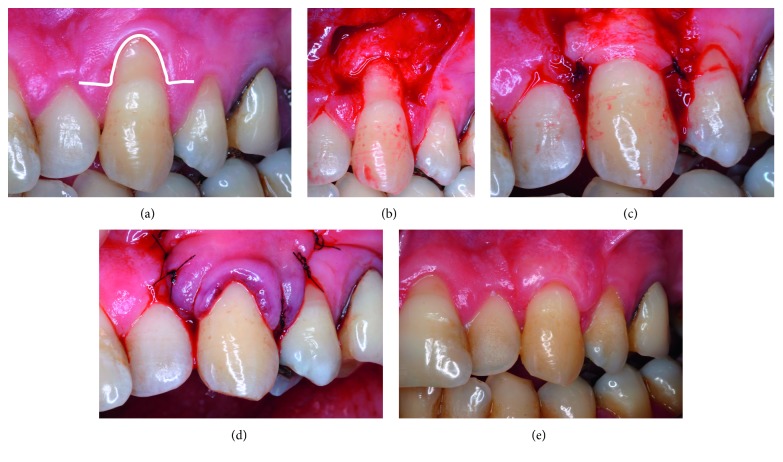
Clinical images illustrating the CAF + CTG technique used. (a) Line shows design of horizontal incisions at CEJ level and sulcular incision; (b) vertical releasing incisions resulting in a trapezoidal flap; (c) CTG secured at CEJ level by interrupted single sutures; (d) CAF sutured 1 mm coronal to CEJ; (e) 6-month follow-up. For abbreviations, see Figure 1 caption.

*CAF* *+* *CTG* (Figure 3) is based on the original trapezoidal flap design of Langer and Langer [26]. Horizontal incisions were performed, mesial and distal to CEJ, leaving the papillae intact and were connected by a sulcular incision on the buccal aspect of the defect (Figure 3(a)). Vertical releasing incisions were then performed, delineating the trapezoidal flap (Figure 3(b)). Full-thickness elevation was performed to the MGJ followed by split-thickness elevation (sharp dissection (15C scalpel blade, Swann-Morton, Sheffield, England)) apical to MGJ (Figure 3(b)). Papillae were deepithelialized and appropriately sized CTG (see above section) was positioned at CEJ and secured by one interrupted suture (Vycril 6–0, Ethicon©, Johnson and Johnson, São José dos Campos, SP, Brasil) at each papilla (Figure 3(c)). Trapezoidal flap was then advanced to 1 mm coronal to CEJ and secured with suspensory suture (Figure 3(d)). Vertical incisions were sutured using interrupted sutures (Figure 3(d)).

**Figure 4:**
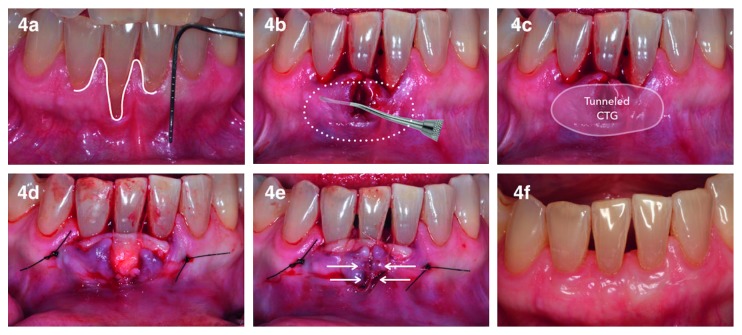
Clinical images illustrating the DPE + CTG technique used. (a) Line represents the design of sulcular and papillary incisions; (b) dotted line represents flap extension; (c) area highlighted by line and light white shading represents CTG position; (d) CTG positioned and stabilized by sutures (single interrupted suture at each end and sling suture at CEJ level; (e) single interrupted sutures used to approximate papillae (arrows); (f) 6-month follow-up. For abbreviations, see Figure 1 caption.

*DPE* *+* *CTG* (Figure 4) is based on the envelope flap design [27] modified to include elevation and approximation of adjacent papillae [28, 29]. A sulcular incision was made first on the buccal aspect of the defect, extending to both adjacent papillae (Figure 4(a)). Subsequently, a full-thickness flap was reflected to create an envelope extending 5 mm apical to RD and encompassing the two adjacent teeth (Figure 4(b)). Appropriately sized CTG (see above) was placed in the envelope (Figure 4(c)), positioned at CEJ level whenever possible and stabilized by two interrupted sutures, one at either end of the envelope flap and a suspensory suture around the defect site tooth (Figure 4(d)). Subsequently, interrupted sutures, spaced 2 mm apart, were used to approximate the mesial and distal margins of the recession defect starting at apical end and finishing with a suture connecting the 2 elevated papillae (Figure 4(e)). Lastly, a suspensory suture penetrating the flap 3 mm apical to GM (of the adjacent tooth), at the level of the proximal aspect of each adjacent tooth, was applied to stabilize the flap (Figure 4(e)).

**Figure 5:**
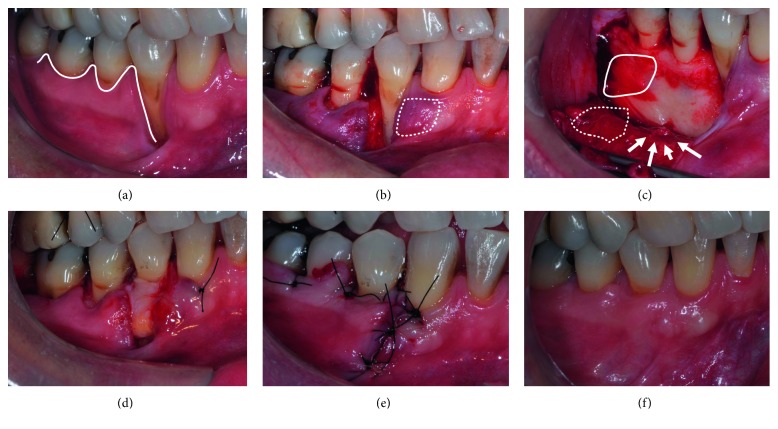
Clinical images illustrating the LSF + CTG technique used. (a) Line represents sulcular and papilla incision design; (b) dotted line outlines tunneled region that will receive CTG; (c) combined full- and split-thickness flap elevated. Continuous line outlines split-thickness portion and dotted line indicates where releasing incision can be performed. Area of mental nerve emergence is noted (arrows), where incision must be avoided. (d) CTG placed in pouch and secured by interrupted sutures at edge and at papilla region. Note also the deepithelialized region (pouch margin) that will receive LSF; (e) LSF, covering CTG and recipient bed, secured by sling suture at recession area and single sutures in the remaining portions. (f) 6-month follow-up. For abbreviations, see Figure 1 caption.

*LSF* *+* *CTG* (Figure 5) is based on the original LSF design [24, 25, 30] combining elements of the pouch approach [31]. Full-thickness flap including papillae was elevated on the distal of the defect up to the distal of adjacent distal tooth (Figure 5(a)). This was followed by a tunnel preparation on the opposite (mesial) defect aspect, i.e., in the area of the adjacent mesial tooth (Figure 5(b)). Subsequently, a movable flap (LSF) covering 2-3 teeth was created by performing a releasing incision on the elevated flap starting at the distal of adjacent distal tooth (Figure 5(c)). Split-thickness elevation may be performed apical to MGJ to provide additional flap mobility (Figure 5(c)). When potentially encroaching on the mental foramen, releasing incisions are placed distal to the foramen (Figure 5(c)). Appropriately sized CTG (see above) was positioned inside the prepared tunnel mesially with the remaining portion covering the defect area. CTG was stabilized by two simple interrupted sutures, one on the prepared tunnel and one on the opposite papilla of the defect site (Figure 5(d)). The LSF recipient site (marginal area of prepared tunnel) was deepithelialized (Figure 5(d)), and the mobilized flap was then laterally positioned and secured with suspensory sutures (one per tooth; to immobilize flap) and simple interrupted sutures (to approximate defect margins and secure LSF margin on recipient site) (Figure 5(e)).

*CTG harvesting.* CTG was harvested using the parallel incisions technique [29]. Following harvesting, a collagen sponge (Hemospon, Technew, Rio de Janeiro, RJ, Brasil) was placed at the donor site and the wound was sutured.

### 2.6. Postoperative Protocol

Dressing was not applied. Patients were instructed to stop all mechanical plaque control in the surgical area for 3 weeks. They received prescriptions for antimicriobial rinse (chlorhexidine gluconate 0.12%, 60 seconds twice daily, 21 days) and analgesic (paracetamol 750 mg, 4x/day, 3 days). Donor and recipient site sutures were removed at 7 and 21 days, respectively. Thereafter, a postsurgical toothbrush was dispensed, to be used at the treated area for the next 20 days. Subsequently, patients were instructed to resume regular soft toothbrush use (Stillman's modified technique).

### 2.7. Statistical Analysis

Normality of the data (Kolmogorov–Smirnov test) and homogeneity of variances (Levene test) were confirmed before further analysis. Descriptive data were recorded as mean ± standard deviation (SD). Paired Student's *t*-test was used to compare baseline values and 6-month outcomes. Paired *t*-test was used to verify intragroup RD changes. Independent samples *t*-test was used to compare different Miller Class defects (II or III) and different RW (narrow or wide) regarding %RC, GR reduction, and KTW change. Pearson correlation coefficient (PCC) was used to measure correlation between GR reduction and baseline RD or RW. Statistical significance was set at *p* < 0.05.

## 3. Results

### 3.1. Study Population

Records of sixteen (11 females) healthy nonsmoking adults, aged 41.6 ± 10.8 years (range: 24–57 years), met the inclusion criteria. Each patient had a single deep GR treated. Of the 16 (10 mandibular) treated teeth, 14 were anterior (8 mandibular), and two were posterior (mandibular premolar and molar). Nine defects were Miller Class II and 7 were Miller Class III.

### 3.2. Baseline Clinical Parameters

Individual patient data, by surgical approach, for RD, RW, GR reduction and RC are shown in Table 1 and for KTW in Table 2. Baseline RD was 6.7 ± 1.8 mm (range: 5–10 mm) and RW was 4.0 ± 1.1 mm (range: 2.0–5.5 mm) (Table 1), with 9 defects classified as wide. KTW averaged 0.2 ± 0.4 mm (range: 0-1 mm) (Table 2). PD at all surgical sites was ≤3 mm. CAL range was 6–13 mm. All sites were BOP-negative and plaque-free at surgery time.

### 3.3. Clinical Outcomes: Entire Study Population

All procedures were completed uneventfully, and no postoperative complications were noted or reported by patients during the early (≤1 month) healing period. During subsequent follow-up, all patients reported that their chief complaint was resolved.

At 6 months postoperatively, all sites were BOP-negative and presented PD ≤ 3 mm. RD was 1.2 ± 0.8 mm (range: 0–2.5 mm), significantly different from baseline (*p* < 0.05). GR reduction was 5.5 ± 2.0 mm (range: 3–10 mm) and RC was 81.7 ± 13.0% (Table 1); two sites had complete RC. Miller Class II defect RC (87.1 ± 9.2%; range: 70.6–100%) compared to Miller Class III defect RC (74.6 ± 14.5%; range: 61.5–100%) approached but did not reach statistical significance (*p*=0.07). No significant differences in RC (*p*=0.21) or GR reduction (*p*=0.80) were detected when comparing narrow (86.4 ± 13.0%; 5.6 ± 1.6 mm) versus wide defects (78.0 ± 12.6%; 5.4 ± 2.4 mm) at 6 months.

Regarding KTW, a significant increase was noted from baseline to 6 months (Table 2). GR reduction was strongly and positively correlated with baseline RD (*r*=0.91; *p* < 0.0001).

### 3.4. Clinical Outcomes by Surgical Approach

*CAF* *+* *CTG*. Individual patient (cases 1, 2, 3, 4, and 5) and group data are presented in Table 1.  Figure 3(e) illustrates the 6-month follow-up of a case treated with this technique.

*DPE* *+* *CTG.* Individual patient (cases 6, 7, 8, 9, 10, and 11) and group data are presented in Table 1. Figure 4(f) illustrates the 6-month follow-up of a case treated with this technique.

*LSF* *+* *CTG*. Individual patient (cases 12, 13, 14, 15, and 16) and group data are presented in Table 1.  Figure 5(f) illustrates the 6-month follow-up of a case treated with this technique.

## 4. Discussion

This retrospective case series documented clinical outcomes when a novel clinical decision-making algorithm was followed to treat single deep (≥5 mm) GRs. The use of this concept resulted in >80% RC and >5 mm GR reduction in Miller Class II and III GR defects with mean baseline RD > 6.5 mm. These findings suggest that deep GRs can be treated with predictability similar to shallow and moderate GRs when using CTG [7] if the proposed surgical approaches are followed. To the best of our knowledge, this is the first study that proposes different flap designs, taking into consideration defect anatomy, GRs ≥5 mm deep, and the first study on outcomes of LSF + CTG.

The lack of studies on treatment of ≥5 mm deep GRs along with the evidence of poorer RC outcomes with increasing RD [7], makes it challenging for practitioners to treat such defects. Similarly, despite evidence that treatment of Class III defects can achieve good results [7], treatment of a Class III deep GR is often fraught with challenges. Use of the proposed algorithm, which aimed to facilitate decision-making and improve treatment predictability for challenging defects, allows a more detailed assessment of relevant anatomical factors, including residual vestibular depth; the latter has not been previously objectively included in a decision tree. Consistent with the evidence of the best RC outcomes, even in Class III defects, when using CTG [7], the algorithm guides the use of CTG-flap approaches that support CTG nutrition and flap stabilization. The biological basis for these choices may have favored the positive study outcomes.

Although there is no directly comparable study (different CTG-flap combinations, GR defects ≥5 mm deep) available, some literature data allow for limited comparisons with the present results. In a study comparing bilaminar technique versus GTR in Miller class I and II GRs, the CAF + CTG group had 18 patients with recessions ≥5 mm (mean baseline RD = 5.6 mm), and achieved 93.5 ± 8.6% RC [20]. Differences in outcomes may be partly explained by inclusion of Class III GRs, absence of Class I GRs, deeper mean baseline RD (6.7 mm), and inclusion of mostly mandibular teeth in the present study. In a case series treating single and multiple GRs using DPE + CTG, Nelson [28] treated 20 teeth with deep RD (range: 7–10 mm) and achieved 88% mean RC. Although Nelson's results are similar to the present study results, inclusion of multiple defects and lack of Miller Class information limit comparability between studies. Present study limitations include the retrospective, nonrandomized design, and the small sample size per individual surgical technique; the latter precluded analysis of intergroup differences in outcomes.

According to a meta-analysis of individual patient data by Chambrone et al. [32] including 320 patients from 22 trials the mean baseline RD for included defects was 3.3 ± 1.1 mm. This result illustrates that most of RC literature has focused on shallow/moderate defects. Evaluation of baseline data of 43 studies investigating CTG-based procedures included in the Chambrone and Tatakis [7] systematic review corroborates the previous finding; the mean baseline RD for Miller Class I and II defects was 3.3 mm and 2.3 mm for Miller Class III GRs. The corresponding compiled results of these CTG-based studies indicated mean RC of 86.9% and 69.9% for Miller Class I/II and for Class III, respectively [7]; the present study RC outcomes for deep GR defects compare favorably with outcomes obtained in shallow/moderate defects. The lack of evidence evaluating deep (≥5 mm) GRs highlights the need for studies on this clinical scenario. Given the evidence that RD increases with time in untreated GRs, use of a predictable treatment approach could improve the long-term outlook for teeth with such deep GR defects [4].

Despite the extensive evidence on CTG-based approaches, mainly CAF-associated [7], and the availability of few studies on LSF alone [33, 34], the literature lacks studies on LSF + CTG combination. Only two recent case reports using LSF + CTG are available [35, 36]. The present study results suggest that in deep and wide GRs LSF + CTG can provide RC outcomes similar to the ones expected from CAF + CTG in shallow/moderate defects [7].

Comparing the decision-aid model proposed by Bouchard et al. [21] with the present decision tree, there are some critical differences. First, and most important, the algorithm described herein is for single GRs with RD ≥ 5 mm, while the Bouchard model referred to single, multiple, shallow (<3 mm), moderate (3–5 mm) and deep (>5 mm) defects. Regarding deep GRs, Bouchard et al. [21] suggested previously described flap designs, while the present decision tree includes novel flap approaches. In shallow vestibule cases, Bouchard et al. [21] advise use of Envelope + CTG as the main choice, considering LSF a second option for single defects; in deep vestibule cases, both CAF + CTG and Envelope + CTG may be used. Although Bouchard et al. [21] used vestibular depth as an important reference for decision-making, classification of vestibular depth was not addressed. This lack of standardization can lead to subjective decision-making and may hinder translation into practice; the present algorithm is based on proportion of RD in relation to residual vestibule, overcoming this limitation. Bouchard et al. [21] did not consider RW as a decision parameter for flap design choice in deep defects or use of DPE + CTG or LSF + CTG; in contrast, the present decision tree incorporates RW as a determinant for the primary selection between DPE + CTG and LSF + CTG, two techniques at the core of the proposed approach.

The presented algorithm, which represents an initial reference for the practitioner when dealing with similar deep GR defects, was based on biological rationale. Nevertheless, biology is not an exact science, and use of an equation (such as the proposed one) may not adequately fit every case. Prospective investigations are necessary to validate and possibly improve this decision-making approach.

## 5. Conclusions

Following a decision-making algorithm to treat deep gingival recessions resulted in significantly positive root coverage outcomes. The proposed algorithm allows the clinician to select the appropriate surgical technique for treatment of single deep gingival recessions with good predictability.

## Figures and Tables

**Table 1:** Individual patient data and descriptive statistics for RD, RW, GR reduction, and RC.

Flap design	Patient	Tooth	Miller class	Baseline RW (mm)	Baseline RW (mm)	6-month RD (mm)	RD reduction (mm)	6-month RC (%)
CAF + CTG	1	6	III	5	6.5	2	4.5	69.2
	2	11	III	5	6	2	4	66.7
	3	11	II	3.5	5	1	4	80.0
	4	9	III	5.5	5	1	4	80.0
	5	11	II	5.5	5	0.5	4.5	90.0
Mean ± SD				4.9 (±0.8)	5.5 (±0.7)A	1.3 (±0.7)B	4.2 (±0.3)	77.2 (±9.4)

DPE + CTG	6	25	III	3	6.5	2.5	4	61.6
	7	25	II	3	6.5	0.5	6	92.3
	8	26	III	2.5	5	0	5	100
	9	27	II	4.5	5	1	4	80.0
	10	11	II	4.5	10	0	10	100
	11	27	III	4	5	2	3	60.0
Mean ± SD				3.6 (±0.9)	6.3 (±1.9)A	1.0 (±1.0)B	5.3 (±2.5)	82.3 (±18.2)

LSF + CTG	12	25	II	2	7.5	0.5	7	93.3
	13	28	III	4	10	1.5	8.5	85
	14	19	II	5.5	8.5	2.5	6	70.6
	15	25	II	3.5	9	0.5	8.5	94.4
	16	25	II	3	6	1	5	83.3
Mean ± SD				3.6 (±1.3)	8.2 (±1.5)A	1.2 (±0.8)B	7 (±1.5)	85.3 (±9.6)

Overall mean ± SD				4.0 (±1.1)	6.7 (±1.8)A	1.2 (±0.8)B	5.5 (±2.0)	81.7 (±13.0)

RD = recession depth; RW = recession width; GR = gingival recession; RC = root coverage; CAF = coronally advanced flap; DPE = double papilla envelope; LSF = lateral sliding flap; CTG = connective tissue graft; different upper cases indicate statistically significant intragroup differences.

**Table 2:** Individual patient data and descriptive statistics for KTW.

Flap design	Patient	Tooth	Miller class	Baseline KTW (mm)	6-month KTW (mm)	KTW change (mm)
CAF + CTG	1	6	III	0	2	2
	2	11	III	0	2	2
	3	11	II	0.5	1	0.5
	4	9	III	1	4	3
	5	11	II	1	3	2
Mean ± SD				0.5 (±0.5)A	2.4 (±1.1)B	1.9 (±0.9)

DPE + CTG	6	25	III	0	4	4
	7	25	II	0	3	3
	8	26	III	0	5	5
	9	27	II	0	3	3
	10	11	II	0.5	7	6.5
	11	27	III	0	3.5	3.5
Mean ± SD				0.1 (±0.2)A	4.3 (±1.5)B	4.2 (±1.4)

LSF + CTG	12	25	II	0	3.5	3.5
	13	28	III	0	4	4
	14	19	II	0	2	2
	15	25	II	0	5	5
	16	25	II	0	3	3
Mean ± SD				0 (±0)A	3.5 (±1.1)B	3.5 (±1.1)

Overall mean ± SD				0.2 (±0.4)A	3.4 (±1.4)B	3.3 (±1.5)

KTW = keratinized tissue width; CAF = coronally advanced flap; DPE = double papilla envelope; LSF = lateral sliding flap; CTG = connective tissue graft; different upper cases indicate statistically significant intragroup differences.

## Data Availability

The data used to support the findings of this study are included within the article.
